# Cell-Mediated Responses to Human Metapneumovirus Infection

**DOI:** 10.3390/v12050542

**Published:** 2020-05-14

**Authors:** Marlies Ballegeer, Xavier Saelens

**Affiliations:** 1 VIB-UGent Center for Medical Biotechnology, VIB, B-9052 Ghent, Belgium; marlies.ballegeer@vib-ugent.be; 2 Department of Biochemistry and Microbiology, Ghent University, B-9000 Ghent, Belgium

**Keywords:** human metapneumovirus, innate and adaptive immune response, evasion strategies

## Abstract

Viruses are the most common cause of acute respiratory tract infections (ARTI). Human metapneumovirus (hMPV) frequently causes viral pneumonia which can become life-threatening if the virus spreads to the lungs. Even though hMPV was only isolated in 2001, this negative-stranded RNA virus has probably been circulating in the human population for many decades. Interestingly, almost all adults have serologic evidence of hMPV infection. A well-established host immune response is evoked when hMPV infection occurs. However, the virus has evolved to circumvent and even exploit the host immune response. Further, infection with hMPV induces a weak memory response, and re-infections during life are common. In this review, we provide a comprehensive overview of the different cell types involved in the immune response in order to better understand the immunopathology induced by hMPV. Such knowledge may contribute to the development of vaccines and therapeutics directed against hMPV.

## 1. Introduction

Acute respiratory tract infections (ARTI) are the most common cause of symptomatic illness worldwide. Although some bacteria and fungi can cause ARTI, viruses are by far the most common cause of ARTI [1,2]. ARTI that are confined to the upper respiratory tract typically result in mild respiratory symptoms. However, when the infection spreads to the lungs, this can lead to life-threatening pneumonia. Two members of the Pneumoviridae family, namely, human respiratory syncytial virus (hRSV) and human metapneumovirus (hMPV), frequently cause viral pneumonia in infants and children (<five years of age), the elderly (>65 years of age), and immune-compromised individuals [3,4,5]. hRSV, first isolated in 1956 from a colony of chimpanzees [6], is now estimated to be the most common cause of childhood pneumonia worldwide [7]. hMPV, first isolated from children in the Netherlands [3], is an important cause of bronchiolitis and pneumonia in children[8,9,10,11]. Several studies have shown that up to 95% of children infected with hMPV were previously healthy, indicating that young age is one of the major factors influencing disease severity [12,13]. Hospitalization rates due to hMPV infection are highest in the first five years, with a peak age between six and 12 months of age [12,14,15,16,17,18,19]. Interestingly, a significant fraction of ARTI that was first considered to have an unknown cause is now attributed to infection with hMPV, supporting early observations that hMPV had been circulating in the human population long before it was first isolated [3]. Supporting that, nearly 100% of people test positive for antibody reactivity in their blood by the age of 10, and almost all adults have serologic evidence of prior hMPV infection [3,20,21,22]. hMPV is classified into two major genetic lineages, hMPV A and B, that are further subdivided into lineages A1, A2, B1, and B2 [3,23,24]. The circulation of the four genetic lineages of hMPV was confirmed in worldwide studies. Long-term retrospective studies conducted in the United States from 1981 to 2001 concluded that multiple lineages can circulate in the same period at a given location [25,26]. Co-circulation of both hMPV A and B genotypes has been documented both in children [27] and adults [28]. However, generally one lineage dominates a season, which varies year by year [29,30]. Studies in rodents and non-human primates show a high degree of cross-protection and -neutralization between different hMPV lineages [31]. However, studies using lineage-specific antisera of ferrets and Syrian golden hamsters have shown that homologous virus-neutralizing titers were significantly higher than titers against heterologous hMPV lineages and that the antigenic relatedness between viruses from two genetic lineages was relatively low [32,33]. These observations of limited cross-protection, together with reports of re-infections of macaques [34] and humans [35] with genetically distinct hMPV strains, might explain why it is possible that multiple lineages of hMPV can co-circulate.

**Figure 1:**
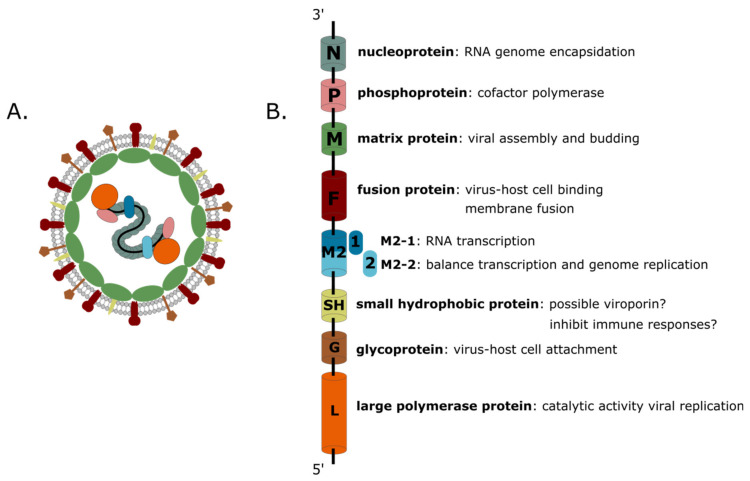
Human metapneumovirus (hMPV) virion structure with viral proteins and their function. Schematic representation of the hMPV viral particle (**A**) and viral genome with encoded proteins (**B**): nucleoprotein (N), phosphoprotein (P), matrix protein (M), fusion protein (F), matrix-2 proteins (M2-1 and M2-2), small hydrophobic (SH) protein, glycoprotein (G), and large (L) polymerase protein.

hMPV is an enveloped negative-stranded RNA virus with a non-segmented genome of approximately13.3 kilobases. The viral genome comprises eight genes and codes for nine proteins: nucleoprotein (N), phosphoprotein (P), matrix protein (M), fusion protein (F), matrix-2 proteins (M2-1 and M2-2), small hydrophobic (SH) protein, glycoprotein (G), and large (L) polymerase protein (Figure 1). Together, the N, L, and P proteins form the viral replication complex. Interestingly, the gene order of hMPV is not only different from that of hRSV, but the virus also lacks the non-structural proteins NS1 and NS2 [3]. Three transmembrane surface glycoproteins are embedded in the lipid envelope: F, G, and SH. The G protein is important for the attachment of the virion to the host cell. The F protein mediates fusion of the viral and host cell membrane. The exact function of the SH protein remains elusive. The F protein sequence is relatively well-conserved between different hMPV genotypes compared to G and SH which are more variable [24,32,36,37]. In addition, when grafted into the genome of recombinant replication competent human parainfluenza viruses that were subsequently used to infect hamsters, hMPV F, but not G or SH, was shown to be able to induce neutralizing antibodies [38,39]. In the ribonucleoprotein complex, the viral RNA is entirely coated by the N protein, resulting in flexible helical nucleocapsids, which are also decorated with the P protein and can recruit the L and M2-1 proteins. In negative-stranded RNA viruses, the viral nucleocapsid serves to protect the viral RNA from degradation and as a template for viral replication and transcription [40].

In general, when respiratory viruses break through the mucosal layer that lines the airway epithelial cells (AECs), dendritic cells (DCs) may already come into play, because dendrites of these cells can sample particles that are present in the airway lumen. DCs that take up virions, for example, can process viral proteins into peptides for antigen presentation. Upon DC migration to the regional draining lymph node, the processed viral antigen can be presented to CD4+ and CD8+ T cells. The activated effector T cells with a matching T cell receptor subsequently expand and travel to the lung parenchyma where they can eliminate the infected epithelial cells [41].

This well-established immune response is also crucial for hMPV clearance and antiviral defense, so when the immune system is compromised, hMPV can persist as has been observed in hematopoietic stem cell recipients [42,43]. Interestingly, hMPV has developed several mechanisms to circumvent the immune system (reviewed here and in [44]). hMPV infection induces a rather weak immune memory response, which helps to explain why recurrent infections during life are common [35,45,46]. Here, we provide a comprehensive overview of the different cell types involved in the immune response against hMPV infection and some new insights into the strategies the virus has developed to evade the host immune response.

### 1.1. Airway Epithelial Cells (AECs)

The major cell populations that can sense hMPV in an acute infection are AECs [47], alveolar macrophages (AMφ) [48], and DCs [49]. hMPV primarily replicates in the epithelial cells located in the upper (nasopharynx) and lower (lungs) respiratory tract [25,26,50,51,52]. The infection gives rise to airway epithelial injury and remodeling [53,54,55]. AECs are armed with pattern recognition receptors (PRRs) that can recognize pathogen-associated molecular patterns (PAMPs) such as viral RNA. PAMP recognition triggers signaling cascades that lead to transcriptional upregulation of cytokines and chemokines and regulate the inflammatory responses in the infected host [56]. In general, two major PRR pathways have been described that can recognize different viral products: (i) the RIG-I-like receptors (RLRs) retinoic-acid inducible gene I (RIG-I) and melanoma differentiation-associated 5 (MDA-5) and (ii) Toll-like receptors (TLRs) including TLR3, TRL4, and TLR7. hMPV infection of A549 cells, a human alveolar type II like epithelial cell line, induces both RIG-I and MDA-5 gene and protein expression [57]. In addition, A549 cells secrete a variety of cytokines and chemokines, as well as type I interferons (IFNs), upon hMPV infection [58]. Interestingly, although hMPV can directly induce RIG-I expression, type I IFNs enhance the induction [57]. Interestingly, RIG-I-dependent signaling, but not MDA-5, is necessary to induce an antiviral state in A549 cells and reduce viral replication. Only inhibition of RIG-I expression significantly reduces activation of interferon regulatory factors (IRFs), transcription factors, and production of type I IFNs [57]. It is important to note that RIG-I gene silencing resulted in incomplete abrogation of cellular responses and that other pathways are also involved. Bao et al. showed that TLR3 expression is induced in AECs upon hMPV infection [59]. However, inhibition of TLR3 expression does not affect hMPV-induced chemokine gene expression in A549 cells [57]. Triggering of RIG-I and MDA-5 leads to the recruitment of a signaling complex to the outer membrane of mitochondria and activation of IRFs and subsequent IFN and interferon-stimulated gene (ISG) expression. Mitochondrial antiviral-signaling protein or MAVS has been identified as an adaptor protein in this signaling complex [60,61] and plays an important role in hMPV-induced signaling in airway epithelial cells [57].

IFNs are potent antiviral cytokines which also have important roles in shaping the adaptive immune response [62]. IFNs can amplify the antiviral response and limit viral replication by inducing the expression of ISGs. Several in vitro studies have shown that different strains of hMPV induce the production of IFNs (type I, II, and III) in A549 cells and that this response relies on viral replication [57,58,59,63,64]. Schoggins et al. have catalogued several ISGs that might have an inhibitory effect on different DNA and RNA viruses [65]. The top hits for hMPV inhibition are CD9, HPSE, P2BY6, and interferon induced transmembrane protein 3 (IFITM3) [65]. Supporting that, McMichael et al. showed that the expression of IFITM3 inhibits hMPV infection in vitro by dysregulation of hMPV F protein-mediated cell to cell fusion [66]. In general, a viral infection induces IFN-dependent antiviral defense strategies in AECs. As a counteraction, viruses modulate this response via several mechanisms resulting in inhibition of IFN synthesis, affecting type I IFN responses and blocking the expression of ISGs [67]. hMPV has been reported to inhibit type I IFN signaling at different levels of the signaling cascade involving the regulation of signal transducer and activator of transcription 1 (STAT1), STAT2, Janus kinase 1 (Jak1), tyrosine kinase 2 (Tyk2), and surface expression of interferon alpha receptor subunit 1 (IFNAR1) [68,69]. In addition, the G protein has been reported to interact with RIG-I and to block RIG-I-dependent gene transcription and thus IFN production [70]. For a detailed overview of the interferon response upon hMPV infection in vitro, animal models and clinical settings, we refer to [71].

Triggering of PRRs does not only induce IFN expression, it also upregulates several pro-inflammatory genes activating the immune response, resulting in pulmonary inflammation and viral clearance. Chemokines are important cytokines that regulate the migration and activation of leukocytes including neutrophil, macrophages, and monocytes to the site of infection [72]. hMPV-infected AECs secrete an important array of pro-inflammatory chemokines carrying CC and CXC motifs [59]. CC-bearing chemokines include CCL2, CCL3, CCL4, and CCL5. CXC-bearing chemokines include CXCL1, CXCL2, CXCL3, CXCL8/IL-8, CXCL10, and CXCL11. CCL2 is an important chemoattractant for monocytes [73] whereas CCL3 and CCL4 recruit and activate neutrophils [74]. In a mouse model of hMPV infection, the majority of lung inflammatory cells are neutrophils and mononuclear cells including macrophages/monocytes during the first days of infection. However, in cotton rats, recruited monocyte/macrophage cell counts were lower [75]. In conclusion, the recruitment of monocytes to the lungs after hMPV infection has been described in hMPV infection animal models, but their importance has not been fully explored.

hMPV also induces expression of thymic stromal lymphopoietin (TSLP) and interleukin 33 (IL-33) in AECs in vitro [76]. Both cytokines activate DCs by inducing the expression of OX40L. TSLP-induced OX40L on DCs primes naïve CD4+ T cells to differentiate into Th2 cells [35,36]. TLSP-activated DCs in turn produce CCL17, an important Th2 enhancing chemokine and attractor of natural killer (NK) cells [77,78]. In lungs of hMPV-infected mice, the expression of TSLP and CCL17 was confirmed. Further studies based on genetic abrogation and immunological blocking of the TSLP-TSLPR pathway revealed that TSLP is one of the main causes of hMPV-induced lung inflammation, and, remarkably, also contributed to hMPV replication in mice [76]. More recently, Li et al. showed that only the pro-inflammatory long form of TSLP (lfTSLP) is induced by hMPV and that the levels of the short form (sfTSLP) with anti-inflammatory functions are not changed [79]. The lfTSLP induction depends on the activation of NF-κB downstream of RIG-I and TLR3 and is facilitated by TANK binding kinase 1 (TBK1) [79]. In conclusion, by activating the TSLP pathway and creating a microenvironment that favors Th2 responses, hMPV might hamper or delay a more efficient antiviral Th1 response [76]. 

One may expect that viral infection of AECs will result in a gene expression profile with an innate immune signature. Indeed, several transcriptomic studies focused on the many upregulated genes that are involved in the initiation of pro-inflammatory and antiviral immune responses, including chemokines, cytokines, type I IFN, and interferon-inducible proteins [59,64,80]. However, other and more recent transcriptomic studies also show that metabolic genes and several mucins are upregulated, and cilium-associated genes are downregulated [63,81]. It is important to note that approximately 20% of the genes affected by hMPV infection are related to metabolic pathways. Moreover, most of the core metabolic enzymes are downregulated upon infection [82]. More specifically, hMPV infection causes tricarboxylic acid (TCA) cycle impairment and drives de novo fatty acid synthesis which has been shown to be necessary for replication of several viruses [83]. Another interesting observation was the fact that hMPV induced a progressive decrease of antioxidant enzyme (AOE) expression levels in AECs and the lungs of infected mice. Reduced AOE expression usually results in increased levels of oxidative stress markers illustrating an imbalance between reactive oxygen species production and antioxidant cellular defenses [59,84]. The transcription factor NF-E2-related factor or Nrf2 regulates antioxidant and cellular protective gene expression, mainly in response to oxidative stress [85]. A recent study suggests that NrF2-dependent genes affect viral replication, airway obstruction, clinical disease, and neutrophilia upon hMPV infection. Using Nrf2 KO mice, Ivancuic et al. showed that functional deficits in Nrf2 and inadequate AOE expression lead to viral-mediated oxidative stress and damage of the airways [86]. 

To limit viral replication and spreading, AECs may undergo programmed cell death or apoptosis to facilitate viral clearance [87,88]. Marsico et al. monitored the behavior of AECs during acute hMPV infection (based on the protocol of Bao et al. [58]) using A549 cells and showed massive cell death characterized by apoptotic nuclei and DNA fragmentation during the first three days and up to seven days post infection (dpi). Moreover, the caspase-3 and -7 activity gradually increased up to 7 dpi, and a role for phosphorylation of the Wee1 kinase in the hMPV-driven apoptosis could be identified [89]. Interestingly, the caspase activity decreased at 14 dpi, and the residual epithelial cells at that time point seem to reverse the apoptotic process. It has indeed been described that prevention of apoptosis in virus-infected cells represents a critical step in establishing viral persistence. Therefore, a persistent virus has developed mechanisms to suppress apoptosis for a longer time to maintain a compartment of infected cells [90]. It is important to note that hMPV persistence has only been described in some mouse studies [54,91,92,93] and a few immunocompromised patients of which some had no symptoms [42,43,94]. Additionally, hMPV persistence has been described in vitro in A549 cells by Marsico et al. [89]. This study showed that at 14 dpi, the hMPV-infected cells were still metabolically active which could represent the beginning of coexistence between AECs and hMPV and thus transition from acute to persistent infections. In general, viruses may be able to persist by interfering with the induction of apoptosis. Supporting that, chronic hMPV infection in AECs increased expression of the anti-apoptotic Bcl-2 protein, which overthrows the apoptotic program [89]. 

### 1.2. Alveolar Macrophages (AMφ)

The resident macrophages of the lungs or alveolar macrophages (AMφ) are localized in the airspaces within alveoli. From this unique position AMφ are able to respond quickly to insults in the lower airways. Under homeostatic conditions, the AMφ are kept in a suppressive state by the lung environment in order to prevent inappropriate inflammatory responses to harmless antigens. However, upon viral infection, the expression of the negative regulators declines rapidly and the lung environment is reshaped. Coupled with the detection of viral antigens and exposure to pro-inflammatory mediators, AMφ adopt a pro-inflammatory phenotype and initiate the innate immune response and viral clearance [95]. AMφ are the primary source of inflammatory and immunomodulatory cytokines in the lungs. They play a pivotal role against both viral and bacterial pathogens and their depletion severely impairs the host response [96,97]. AMφ are the major cellular source of hMPV-induced type I IFNs IFN-α and -β but also other pro-inflammatory cytokines such as tumor necrosis factor alpha (TNF-α) and IL-6 in the lungs. Depletion of AMφ by clodronate-loaded liposomes (L-CL2MBP) before hMPV inoculation enhanced viral replication, pulmonary inflammation, and airway disease [48]. Interestingly, treatment with L-CL2MBP after hMPV infection did not alter lung viral replication (Table 1). The results of these depletion studies imply that AMφ are critical in the early phase of viral entry and that the virus directly targets AMφ. This hypothesis was supported by the fact that hMPV is able to replicate in AMφ isolated from bronchoalveolar lavage (BAL) fluid, ex vivo, although the infection is less efficient than in AECs [48]. A recent study by Li et al. shows that infection with hMPV downregulates C/EBPα, a transcription factor critical for the expression of the homeostatic effector molecule cyclic adenosine monophosphate or cAMP. The suppressive effect on cAMP could be one of the events in AMφ reprogramming in response to hMPV infection [98].

### 1.3. Neutrophils

One of the first type of cells to arrive at the site of respiratory virus infections are neutrophils that specialize in the elimination of infected cells and clearance of microbial pathogens, dead cells, and debris [95]. In addition to their cytotoxic function, they also play a critical role in the innate and adaptive immune response by initiating crosstalk between resident and recruited immune cells including DCs and T cells. The secretion of cytokines and chemokines but also MHC molecules initiates this pivotal crosstalk [104]. It is important to note that, in general, respiratory viruses are not able to infect neutrophils productively. However, these cells can possibly phagocytose virions and apoptotic bodies that contain virus-derived gene products but this has not been described for hMPV [105]. Generally, neutrophils migrate to the site of infection at early time points and their potent but non-specific cytotoxic activity is short-lived. It is important that sufficient neutrophils are recruited in order to control virus dissemination but an excessive neutrophil response can be detrimental for the host. Therefore, neutrophils undergo apoptosis, and the apoptotic bodies and debris they leave behind are cleared by macrophages [95].

The fast influx of neutrophils upon hMPV infection has been well documented in animal studies. In mouse BAL fluid, a significant neutrophilia was observed within the first three days of infection. Moreover, neutrophil counts peaked on day one to two post-infection but decreased gradually starting on day four until day seven (reviewed by Cheemarla et al. [106]). Similar to what is seen in animal studies, hMPV infection is associated with neutrophil influxes in the airways of infected infants. Moreover, high cytotoxic neutrophil infiltration and high amounts of their degranulation products were strongly associated with an increased airway tissue injury [107]. The high neutrophil numbers positively correlated with the high expression levels of chemokines including KC (in mice [75]) and IL-8 (in infants [20]). Interestingly, in a first study where depletion of neutrophils was achieved by an anti-mouse Ly6G-specific monoclonal antibody [108], both inflammation and, surprisingly, lung viral loads were significantly reduced (Table 1) [76]. These results suggest that the recruitment of neutrophils in the airways supports viral replication albeit via an unknown mechanism. In contrast, a more recent study, with the same monoclonal antibody, showed an increased pulmonary inflammation and more severe clinical disease in neutrophil-depleted mice [99]. In this study, neutrophils did not contribute to lung viral clearance since no difference was observed in lung viral titers in the presence or absence of neutrophils (Table 1) [76]. The different outcomes of the two mouse studies can be explained by the difference in the administration scheme of the anti Ly6G antibody or the different hMPV strains used for infection. It is also important to note that during severe lung inflammation, a heterogeneous neutrophil population with distinct activation profiles and different effects on T cells characterize the systemic and local responses [109]. Therefore, one subset of mature neutrophils can clear infection and activate the immune system whereas others induce immunosuppression [110,111]. 

During hMPV infection, the G protein contributes to the recruitment of neutrophils. The exact mechanism still needs to be unraveled but potentially involves the inhibition of IFN responses by the G protein [112]. The G protein has an inhibitory effect on RIG-I mediated activation of IFN-α and IFN-β production [69,70] and inhibits the production and activation of IFN-λ [113]. Inhibition of IFN signaling would lead to higher levels of chemokines and higher neutrophil numbers in the lung. Indeed, infection with recombinant hMPV that lacks the G gene is associated with a reduced expression of TNF-α and IL-17, both known to promote expression of neutrophil chemotactic cytokines [112]. Although it is not known if other hMPV proteins have an effect on neutrophil function, it is likely that proteins that interfere with the IFN pathway such as the SH and M2-2 proteins will have an impact on neutrophil recruitment.

### 1.4. Dendritic Cells (DCs)

Respiratory tract DCs are present within the airway epithelium, submucosa, and lung parenchymal tissue under normal, resting conditions. They are highly likely to encounter virions because of their strategic location at the site of virus entry where they can sample antigens throughout the upper and lower respiratory tracts [114]. Upon infection, DCs can be activated directly by the virus through PRRs or indirectly by pro-inflammatory chemokines and cytokines released by AECs or other resident immune cells [115]. Activated DCs can migrate to the draining lymph nodes where they activate the adaptive immune response or they can stay in the lungs and promote immune responses locally. Plasmacytoid DCs (pDCs) play an essential role in the initial sensing of viral pathogens and initiation of immune response predominantly via the production of type I IFNs. Conventional DCs (cDCs) are mostly associated with the lung epithelium and migrate to the draining lymph nodes in the first days after infection where they function as antigen presenting cells (APCs) [95]. 

The initiation of the immune response via the secretion of IFNs, chemokines, and other cytokines but also the upregulation of a variety of costimulatory molecules and receptors or cell maturation is crucial for priming virus-specific CD4+ T cells and to initiate the antiviral response. Two groups have shown that hMPV can infect both cDCs and pDCs [49,114,116,117]. Although hMPV infection of DCs results in the expression of viral proteins, no major cytopathic effects such as syncytia formation or massive apoptosis were observed [49]. In addition, hMPV infection in both DC subtypes failed to induce a limited production of IFN-α [117]. Both TLR7 and MyD88 are essential for the secretion of type I IFNs by hMPV-infected pDCs [118,119]. Importantly, hMPV infection in DCs also induces the expression of several microRNAs (miRs) such as hsa-miR-182-5p and hsa-miR-4634. Although several microRNAs are believed to be known regulators of DC functions, including maturation, antigen presentation, and cytokine production, no link between the target genes of the hMPV-induced miRNAs and DC function has been found [120,121].

Despite the fact that hMPV-infected DCs showed a mild upregulation of co-stimulatory molecules on their surface, they fail to activate specific naïve T cells efficiently [114,122,123]. Priming of hMPV-specific CD4+ T cells by DCs is a critical process in the induction of an antiviral response so naturally, hMPV seems to have developed several mechanisms to impair these functions of DCs [116,123]. 

To maintain efficient virus growth, hMPV has developed different strategies to minimize host IFN production. It is important to note that some of the studies described gave conflicting results, and there is still no consensus about the interactions between hMPV proteins and different host factors associated with the immune response. When investigating activation of IFN pathways upon hMPV infection, it is important to keep in mind that in vitro passage of hMPV may lead to the accumulation of so-called defective interfering (DI) particles [124]. DIs arise spontaneously when virus are passaged at high multiplicity of infection (m.o.i). in mammalian cell lines due to errors made by the replicase complex and consist of virions with partially deleted viral genomes [125]. Therefore, the presence of DIs could possibly explain the discrepancy in the reported results concerning IFN induction.

The G and SH proteins have been considered for a long time as non-essential proteins for hMPV replication, illustrated by the observation that recombinant hMPV viruses lacking either G, SH or both genes can replicate efficiently in vitro and in vivo [80,126,127,128]. However, Le Nouën et al. showed that DCs infected with hMPV mutants that lack both the SH and G genes had an increased maturation rate and were able to prime naïve T cells [122]. In addition, Kolli et al. suggested that G promotes the inhibition of TLR4 signaling in DCs, which affects type I IFN secretion [129]. The G protein has also been reported as an interferon antagonist in AECs by Bao et al. [70]. Unfortunately, using siRNA against the G gene, Preston et al. were unable to confirm these results [130], so the question if the G protein interferes with the IFN response remains unanswered for AECs. One group showed that hMPV uses the SH protein to interfere with the TLR7/MyD88 pathway, resulting in the inhibition of type I IFN induction in pDCs [118,131]. Supporting an inhibitory role for SH, Hastings et al. showed that transient expression of the hMPV SH protein was able to prevent IFN-induced STAT1 phosphorylation in Vero cells. Additionally, infection with a recombinant hMPV strain lacking SH resulted in phosphorylation of STAT1 at the same level as uninfected cells [132]. In contrast, de Graaf et al. were unable to identify any function of SH in the context of viral replication or immune response in vitro in the host cell using an hMPV mutant lacking SH [80]. In addition, infection with recombinant hMPV that lacks SH presented no difference in viral kinetics and pathogenesis in a rodent model [126] and minimal attenuation in non-human primates [133]. In addition to the well-studied G and SH proteins, other viral proteins have been described to mediate hMPV immune evasion. A study by Ren et al. indicated that DCs infected with an hMPV mutant lacking the M2-2 protein produce higher amounts of MyD88-dependent genes including cytokines, chemokines and type I IFNs [134]. More specifically, M2-2 blocks the TLR7/9 dependent signaling pathway by suppressing IRF7 homodimerization through partial inhibition of IRF7 phosphorylation [135]. Two putative PDZ binding motifs in M2-2 play a major suppressive role in cellular innate immunity both in vitro and in vivo [136,137]. Infection of mice with reverse engineered hMPV virus with mutations in the M2-2 PDZ-binding motif induced higher DC maturation and recruitment of DCs and T cells in the lungs. These in vivo results highlight the potential for modification of M2-2 PDZ-binding motifs in order to overcome poor mucosal innate immunity upon hMPV infection [138].

### 1.5. Natural Killer Cells (NKs)

Natural killer (NK) cells respond to viral infection mainly by killing virus-infected cells but also by mediating the adaptive immune response. NK cells are perfectly equipped to distinguish normal cells from virus-infected cells. They express a specialized repertoire of both inhibitory and activating receptors regulating their activity. A large family of inhibitory receptors is able to recognize self-ligands such as MHC class I. In contrast, the number of different NK-activating receptors is limited. In humans, the most important activating NK cell receptors are NKp46, which belongs to the natural cytotoxicity receptors (NCR), and the NKG2D receptor that interacts with stress-induced ligands such as MICA and MICB [139,140]. NKp46 is the only NCR member that has a mouse orthologue named Ncr1. In addition to their direct cytotoxic effect via the release of cytotoxic granules, NK cells also increase cytotoxic T lymphocyte (CTL) activity via the production of IFN-γ [141].

The role of NK cells in the antiviral response against hMPV infection appears to be mouse strain dependent. In both BALB/c and C57BL/6 mouse strains, NK cells are recruited to the lungs upon hMPV infection [91,92,100,142]. Moreover, the recruited NKs are activated, as indicated by the increased release of cytotoxic granules [100,142]. However, depletion of NK cells in BALB/c mice increases the lung viral titers [91] but no effect on viral titers has been seen in C57BL/6 mice (Table 1) [100]. It is important to note that NK1.1 antibody treatment depletes not only NK cells but also NKT cells. NKT cells embody a mixed population of T cells that possess killing properties of both NK and T cells and form a bridge between the innate and adaptive immune system. During viral infections, NKT cells exert effector functions but also regulate immune responses in order to limit lung inflammation [41]. Interestingly, a recent study by Gaya et al. showed that NKT cells promote B cell immunity during viral infections via the release of IL-4 [143]. To exclude the fact that the effects with the NK1.1 antibody might be due to NKT cell depletion, CD1d-deficient mice [144] were infected with hMPV. These mice lack NK1.1+ NKT cells but have normal NK cell numbers and have similar viral titers as NK1.1 antibody-treated mice upon hMPV infection (Table 1) [100]. Together, the mouse studies performed by Wen et al. exclude a role for NK and NKT cells in HMPV infection [100]. 

Upon hMPV infection, the expression of ligands for the activating receptors NKp46 and NCR1 increased [145]. Surprisingly, hMPV proteins do not appear to be recognized by the activating receptors and it remains unclear what the ligand of hMPV-infected cells is. As it is the case for other immune cells of the innate system, hMPV presumably benefits from the avoidance of recognition by NK cells. One possible mechanism is through downregulation of the stress ligand MICA, one of the major ligands of the activation receptor NKG2D and via upregulation of MHC class I, a ligand of the inhibitory receptor [145]. It is also likely that both the G and SH proteins of hMPV inhibit the recruitment of NK cells as these proteins affect the profile of chemokines produced after infection. 1.6. T cells

Conventional T cells play an important role in the control of hMPV replication in the lung during both the early and late phase of hMPV infection. When the total T cell population is depleted in mice, increased viral replication in the lung is observed (Table 1) [91]. In mouse models of hMPV, the number of both CD4+ and CD8+ T cells increased on day four and peaked on day six or eight, respectively. Interestingly, the levels were still elevated two weeks after infection [101]. In hMPV-infected mice, depletion of CD4+ and CD8+ T cells resulted in significantly higher body weight compared to control-treated hMPV-infected animals. The effect was most pronounced when CD4+ T cells were depleted, and no further protection was observed when both cell types were depleted simultaneously (Table 1) [101]. Surprisingly, depletion of CD4+ T cells or CD8+ T cells alone reduces lung pathology without affecting the viral load. Depletion of both subsets did result in higher viral titers, showing that both CD4+ and CD8+ T cells are necessary for clearing the primary infection in the lungs. Together, these data show that CD4+ or CD8+ T cells play an antiviral role by themselves, but the two subsets combined are more effective in the eradication of hMPV from the lungs. In order to evade the immune response, hMPV delays CTL responses [92]. In combination with an abnormal T helper response induced by hMPV-infected DCs, this results in poor clearance of the virus in the lungs [92]. 

Over the last years, the interest in the role of non-conventional T cells in immune homeostasis and during infections has increased. Non-conventional T cells, including γδ T cells, differ from conventional T cells in many ways and are important in bridging the innate and adaptive arms of the immune system. Their ability to exert effector functions soon after activation makes them, in contrast to conventional T cells, important in the early phase of infection. Unfortunately, due to their limited receptor diversity, they recognize conserved, non-peptide antigens [146]. Interestingly, when neutrophils were depleted in hMPV-infected mice, no difference in the number of CD4+ T and CD8+ T cells in the lung was observed; only a significant increase in lung γδ T cells was reported [99]. Under normal conditions, low numbers of γδ T cells reside in the lung in close proximity to epithelial mucosa. These cells are ideally located to participate in the first line of defense against infection [90]. After hMPV infection, the number of these non-conventional T cells increased after five days and the levels remained high after 15 days. In addition, TCRδ KO mice showed decreased pulmonary inflammation upon hMPV infection, supporting an immune suppressive role of γδ T cells in the hMPV-induced inflammatory response (Table 1) [99]. 

#### 1.5.1. CD4+ T Cells

Early studies in BALB/c mice reported a Th1 response in the first week after hMPV infection, which controls initial virus replication. At later time points, polarization towards a Th2 immune response occurs and facilitates viral persistence [91,92]. The observed Th1 phenotype in mice correlates with the cytolytic activity of peripheral blood mononuclear cell (PBMCs) isolated from adults infected with hMPV and the predominant Th1 response generated in infected infants [147,148]. More recently, Douville et al. could not distinguish Th1 responses in the first week of hMPV infection in mice and concluded that mixed Th1 and Th2 responses mediate the immune reaction [149]. It is now clear that hMPV induces a complex T cell response, which is not well characterized and might include a mixed Th1/Th2 response. Independent of their phenotype, CD4+ T cells produce an inefficient IFN-γ response when activated with hMPV-infected PBMCs in vitro [149]. The observed impairment of T cell immunity upon hMPV infection is most likely due to impaired functions of hMPV-infected DCs. Indeed, in vitro studies have shown that the SH and/or G proteins reduce CD4+ T cell proliferation in an assay where they are co-cultured with hMPV-infected DCs [122].

A specific subset of CD4+ T cells, called regulatory T cells or Tregs, are master regulators of the immune system and immune homeostasis by engaging multiple mechanisms. Tregs can inhibit effector cell function and proliferation through the expression of inhibitory cytokines such as transforming growth factor beta (TGF-β) and IL-10. In addition, Tregs can adjust DC function via interaction of receptors such as cytotoxic T-lymphocyte-associated protein 4 (CTLA-4) and programmed cell death protein 1 (PD-1) with their ligands [150]. In response to hMPV, Tregs accumulate in the lungs of mice and are activated [102]. The Tregs responding to hMPV are most likely derived from naïve CD4+ T cells and do not have a thymic origin based on the low expression of markers to phenotype thymic Tregs. Interestingly, Rogers et al. demonstrated the importance of timing in the depletion of Tregs in the course of hMPV infection [102]. hMPV infection in FoxP3^DTR^ mice depleted of Tregs resulted in lower peak virus titers due to more effective anti-viral CD8+ T cells responses but was also associated with increased histopathology. It is important to note that the magnitude of the hMPV-specific CD8+ T cell response was unaltered. In contrast, depletion of Tregs immediately before infection by using an anti-CD25 antibody led to impaired DC and CD8+ T cell migration and delayed virus clearance (Table 1) [102]. In addition, absence of Tregs in the priming stage of infection skewed CD4+ T cells towards a Th2 phenotype. However, it was not studied in detail what the implications on virus-specific T cells were. Rogers et al. describes an important role for Tregs in priming the immune response. However, after the immune response has been initiated, Tregs are not essential and can even have detrimental effects [102]. The effect of hMPV infection on Tregs was also visible in a study with IL-17 deficient mice which have a defect in IL-17 production, leading to an immune response that is dominated by FoxP3+ Tregs. hMPV-infected IL-17 KO mice did not have altered lung viral titers or different lung inflammation scores compared to wild type mice [151]. Interestingly, infected IL-17 KO mice had an increased percentage of Tregs compared to mock-treated IL-17 KO mice and reduced percentages of Th1 and Th2 in the BAL fluid. It is important to note that the amount of Tregs between IL17 KO and WT mice was also different in the absence of hMPV. However, no increase after hMPV infection was seen in WT mice, suggesting that the imbalance between Tregs and Th17 worsens upon hMPV infection [151].

#### 1.5.2. CD8+ T cells

Virus-specific CD8+ T cells can selectively eliminate infected cells. Following peak expansion, the CD8+ T cells numbers shrink and a memory population of virus-specific CD8+ T cells remains in the lungs [41]. hMPV infection results in the accumulation of hMPV-specific cytotoxic and IFN-γ positive effector cells in both the airways and lungs seven days post infection. As expected, the presence of CTL activity correlated with the induction of IFN-γ positive CD8+ T cells. A recent study by Tzannou et al. showed that hMPV-reactive T cells exist in the peripheral blood of healthy and immune-compromised subjects, although the levels were low and these specific T cells needed to be expanded first. In addition, they identified a hierarchy of immunodominance based on a number of donors who responded positively to hMPV antigens. It is interesting that the F protein was recognized by 97% of the donors followed by N, M2-1, M, P, L, P, and SH in that particular order of dominance [152].

Despite the fact that CD8+ T cells secrete IFN-γ and kill infected cells in vitro, they fail to clear viruses in the airways in vivo. Studies by Erickson et al. show that CD8+ T cells become exhausted and upregulate PD-1 and fail to respond when restimulated with viral antigens [153]. Upon hMPV infection, PD-1-mediated pulmonary CD8+ T cell impairment occurred rapidly, already by day seven, and continued for several weeks after viral clearance [153]. Exhausted CD8+ T cells upregulate PD-1 in an antigen-dependent manner, so one way to prevent impairment is CD8+ T cell migration outside the infected lung to escape antigen-driven TCR signaling. In a re-infection model of hMPV, using µMT mice that lack B cells, impairment of memory CD8+ T cells was also observed, and the expression of PD-1 was upregulated. Blockade of PD-1 signaling restored CD8+ T cell function in the lung and enhanced hMPV control in both primary infection and re-infection [153,154]. The exhausted phenotype was also observed in IFNAR1 deficient mice but the phenotype was linked to upregulation of another inhibitory receptor, i.e., Tim3, since the expression of PD-1L is driven by IFN signaling [155].

### 1.6. B Cells

An important step in the control of viral infections is the production of virus-specific antibodies by B cells. These antibodies can neutralize, opsonize and inactivate virions or facilitate killing of infected cells. In order to prevent virus dissemination, the spread of infectious virions to neighboring cells needs to be controlled. Neutralizing antibodies can block the activity of viral surface proteins and prevent free virions from invading uninfected cells [95]. hMPV-specific antibodies are produced after the first infection in childhood and are still detectable after 20 years [3,20,21,22]. The predominant antibodies are directed against the F protein of hMPV. There is an inverse correlation between the levels of anti-hMPV antibodies and susceptibility to hMPV infection [156]. It is important to note that the hMPV-specific antibody response cannot effectively clear the virus, and hMPV is able to persist in the lungs, even in the presence of neutralizing antibodies [91,92]. Therefore, control of hMPV is partly mediated by cytotoxic T lymphocytes and NK cells [91]. The humoral response following natural infection is not sufficient to prevent re-infections. The interplay between the humoral and cell-mediated adaptive response is nicely demonstrated by the fact that production of neutralizing antibodies depends on CD4+ T cells since antibody levels are undetectable in mice depleted of CD4+ T cells [101].

## 2. Conclusions

**Figure 2:**
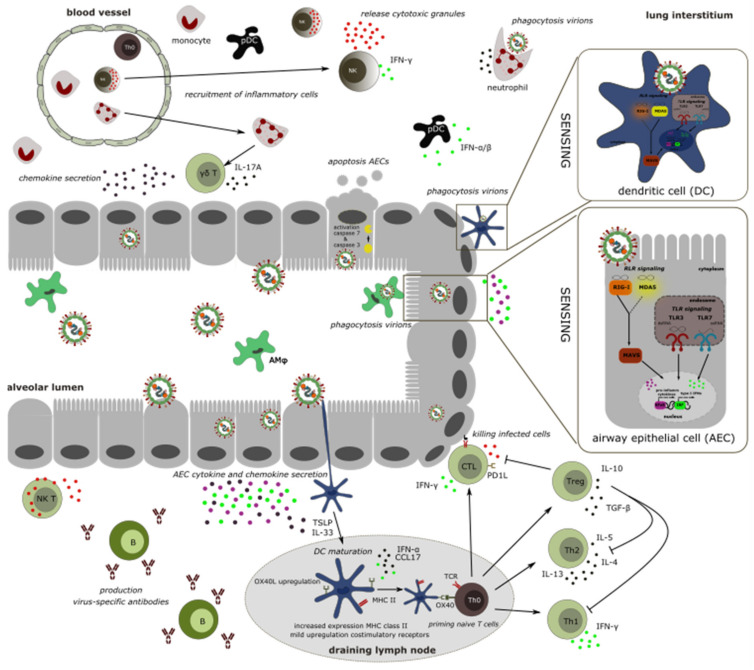
Schematic overview of the interplay between different cell types leading to pulmonary immunopathology upon hMPV infection described in this review. Briefly, dendritic cells (DC), airway epithelial cells (AECs) and alveolar macrophages (AMφ) are the major cell populations that sense hMPV infections. AMφ initiate the primary innate immune response and viral clearance by phagocytosis of hMPV virions. Infected AECs release several pro-inflammatory cytokines, chemokines, and interferons (IFNs) and eventually undergo apoptosis. The signaling pathways induced upon hMPV infection are presented in the zoomed-in box on the right. Activated DCs can stay in the lung and promote local immune responses or they can migrate to the draining lymph nodes and prime naïve T cells (Th0). The activated effector CD4+ and CD8+ T cells expand and migrate back to the lung. Th0 cells can differentiate into several T cell subsets upon hMPV infection including Th1, Th2, regulatory T cells (Tregs), NKT cells, and cytotoxic lymphocytes (CTLs). The last two subsets are able to kill infected cells by the release of cytotoxic granules. Chemokine release by infected AECs recruits several other inflammatory cell types such as NK cells, monocytes, and neutrophils, mediating viral clearance via various killing mechanisms. Another important step in the control of viral infection later in the infection is humoral response through the production of virus-specific antibodies by B cells.

One crucial aspect of the development of vaccines or therapeutics is the knowledge of host immune responses to hMPV infection and understanding of the immunopathology induced by the virus. In this review we focused on the different cell types that are involved in the immune response to hMPV infection and their contribution to hMPV replication and pulmonary inflammation. (Figure 2 and Table 1). Although a plethora of immune cells are involved in the immune response evoked by hMPV infection, the major cell populations that can sense hMPV in an acute infection are AECs, AMφ, and DCs. Because of their unique location, these cells come in first contact, resulting in a first antiviral response characterized predominately by IFN production. Instantly, hMPV tries to counteract the response via several immune repressive mechanisms mainly mediated by its surface proteins G and SH. Since the discovery of hMPV two decades ago, numerous new insights into the interaction between host and hMPV have been described. Unfortunately, there are several studies with conflicting results and up to now the roles of the G and SH proteins as antagonists of the immune responses remain unclear. In the future, it will be necessary to further investigate these interactions in more detail. In addition to mediators of the innate immune system, T cells, both CD4+ and CD8+ T cells, play a crucial role in the clearance of hMPV. Not surprisingly, hMPV has also evolved ways to delay the cytotoxic T cell response and viral clearance by impairing DC cell function. Further, CD8+ T cells become exhausted and upregulate PD-1 and fail to respond when restimulated with hMPV.

In order to be able to replace symptomatic treatment with hMPV-targeted therapies, further research is also needed into the replication cycle of hMPV. For example, a recent report shows that the formation of cytoplasmic inclusion bodies is required for hMPV genome replication and transcription [157]. Additionally, Pan et al. recently resolved the structure of the polymerase phosphoprotein complex [158]. These new observations are crucial in understanding hMPV biology and can lead to the development of new therapeutic strategies. In addition, a vaccine that can prevent disease caused by hMPV infection is highly desirable. Vaccine development for hMPV is, however, challenging because the antibody response induced by natural infection wanes over time [156] and the memory response in the host is weak [149]. Re-infections with hMPV are thus common in both children and adults. Vaccination strategies that merely aim to induce robust virus-specific neutralizing antibody responses should therefore aim to induce long-lasting immunity, which may be achievable with certain adjuvants [159,160]. In addition, the development of a CD8+ T cell-mediated vaccine could be pursued whether or not in combination with the induction of neutralizing antibodies. In order to include CD8+ T cell responses within a future vaccine strategy, more research is definitely needed, especially into the balance between cell-mediated protection and immunopathology. Furthermore, T cell impairment may represent a barrier to vaccination for ARTI, but pharmacologic restoration of T cell function by using, for example, PD-1 blockers, can have detrimental effects in the lungs as well [161].

The human respiratory system is a playground for hMPV. Ultimately, it may take a combination of a directly acting antiviral agent and a host response-modulating drug to control severe disease caused by hMPV.

## Figures and Tables

**Table 1:** Studies showing the importance of different cell types involved in hMPV infection.

Cell Type	Strategy for Cell Type Depletion	Time of Depletion	Lung Viral Load	Lung Inflammation and Histopathology	Mouse Strain	Reference
**Innate** **Immunity**						
AMφ	L-CL_2_MBP liposomes	Before hMPV inoculation	Decreased	Decreased	BALB/c	[48]
		After hMPV inoculation	Unchanged	Not investigated	BALB/c	[48]
Neutrophils	anti-Ly6G monoclonal antibody	Before hMPV inoculation	Unchanged Decreased	Increased Decreased	BALB/c BALB/c	[76] [99]
NK Cells	Anti CD49b/Pan NK cell antibody	Before hMPV inoculation	Increased	Not investigated	BALB/c	[91]
	Anti NK1.1 antibody	Before hMPV inoculation	Unchanged	Unchanged	C57BL/6	[100]
NKT Cells	CD1d-/- mice	Before hMPV inoculation	Unchanged	Unchanged	C57BL/6	[100]
**Adaptive Immunity**						
T Cells	Anti CD4 and anti CD8 antibody	Before hMPV inoculation	Increased	Decreased	BALB/c	[101]
	Anti CD3ε + anti αβTCR antibody	Before hMPV inoculation	Increased	Not investigated	BALB/c	[91]
CD4+ T Cells	Anti CD4 antibody	Before hMPV inoculation	Unchanged	Decreased	BALB/c	[101]
Tregs	FoxP3^DTR^ mice	Throughout hMPV infection	Increased	Increased	C57BL/6	[102]
		Before hMPV inoculation (early)	Increased	Increased	C57BL/6	[102]
		After hMPV inoculation (late)	Unchanged	Increased	C57BL/6	[102]
	Anti CD25 antibody	Before hMPV inoculation	Decreased	Not investigated	C57BL/6	[102]
CD8+ T Cells	Anti CD8 antibody	Before hMPV inoculation	Unchanged	Decreased	BALB/c	[101]
	Adoptive transfer hMPV specific CTLs in Rag1^−/−^ mice	Before hMPV inoculation	Decreased	Not investigated	BALB/c	[103]
γδ T Cells	TCR-δ KO mice (B6.129P2-Tcrd^tm1Mom^/J)	Before hMPV inoculation	Not investigated	Decreased	C57BL/6	[99]

hMPV = human metapneumovirus; AMφ = alveolar macrophages; NK = natural killers cells; NKT = natural killer T; CTL = cytotoxic T lymphocytes; Tregs = regulatory T cells; TCR = T cell receptor; CD = cluster of differentiation; KO = knockout; Rag = recombination activating gene; Ly6G = lymphocyte antigen 6 complex, locus G.
